# Epidemiology and outcomes of tibial plateau fractures involving the medial plateau: a comparative analysis of AO type B and C injuries

**DOI:** 10.1302/2633-1462.73.BJO-2025-0261.R1

**Published:** 2026-03-09

**Authors:** Kischentaran Ravindra Sanmugam, John F. Keating

**Affiliations:** 1 University of Edinburgh, Edinburgh, UK; 2 Department of Orthopaedics, Royal Infirmary of Edinburgh, Edinburgh, UK

**Keywords:** Tibial plateau fracture, Bicondylar tibial plateau, Common peroneal nerve palsy, Compartment syndrome, Postoperative complications, Medial tibial plateau, Infection, Management, Open reduction and internal fixation, Fractures of the tibial plateau, epidemiology, deep infections, comorbidities, Orthopaedic Trauma, compartment syndrome, higher-energy trauma, bicondylar tibial plateau fractures, logistic regression analysis, postoperative complications

## Abstract

**Aims:**

Medial tibial plateau fractures are frequently due to high-energy injuries, and can be difficult to manage and associated with a significant rate of postoperative complications. The goal of the study was to evaluate the epidemiology and results of management of medial tibial plateau fractures, and ascertain the factors influencing its outcomes.

**Methods:**

The patient cohort comprised 143 patients with medial tibial plateau fractures treated over a period of six years. The groups were divided into medial tibial plateau fractures (B-type) and bicondylar tibial plateau fractures (C-type), according to the AO/Orthopaedic Trauma Association (OTA) classification and Schatzker classification. Patient information, including basic demographic details, duration of follow-up, mechanism of injury, comorbidities, management, and postoperative complications, was recorded. Analysis of these data was performed to evaluate outcomes and compare both fracture groups.

**Results:**

Among 143 patients, C-type fractures (43%) were more often linked to high-energy trauma and comorbidities, though not statistically significant (p = 0.051). Both groups were primarily managed with open reduction and internal fixation (ORIF; C-type: 88.5%, B-type: 85.4%, p = 0.582), with 7% managed conservatively. Complications were comparable, but deep infections (13.1% vs 4.9%, p = 0.079) and compartment syndrome (3.3% vs 0%, p = 0.099) were more frequent in C-type. Although C-type fractures demonstrated a trend towards higher complication rates and more complex management, none of the observed differences reached statistical significance. The overall risk of complications did not vary significantly between the two groups (p = 0.639). Logistic regression revealed no significant predictors of fracture type (*R²* = 0.050).

**Conclusion:**

The outcomes of isolated medial tibial plateau fractures are comparable with those of bicondylar tibial plateau fractures, with similar complication rates. Although C-type fractures tended to be associated with higher-energy trauma and increased risks of deep infection and compartment syndrome, these differences were not statistically significant. While medial plateau fractures are often assumed to be less severe, they can be considered injuries of similar complexity to bicondylar patterns.

Cite this article: *Bone Jt Open* 2026;7(3):340–347.

## Introduction

Fractures of the tibial plateau typically occur as a consequence of a combination of valgus deformity associated with axial compression, which most commonly results in lateral tibial plateau fractures. Fractures of the medial plateau are less common, since the varus deforming forces which cause them are less frequently encountered in traumatic injuries. They are more frequently associated with higher-energy trauma producing isolated medial plateau fractures or bicondylar fractures with a medial plateau component. As such, medial tibial plateau injuries are often reported to be difficult to manage, and are subject to higher levels of postoperative complications such as compartment syndrome, neurovascular injury, ligamentous disruption, infection, and post-traumatic arthritis.^1,2^ The Schatzker classification can be used to classify the medial tibial plateau fractures, with type IV being an isolated medial tibial plateau fracture and type V and VI describing bicondylar patterns.^1,3^ The AO/Orthopaedic Trauma Association (OTA) classification is now more commonly used, and provides a scheme for more detailed description of the types of medial plateau fracture encountered.^4^

Although a well-recognized entity, little has been written about medial tibial plateau fractures. The primary focus of this study was to evaluate the results pertaining to the management of medial and bicondylar tibial plateau fractures with a medial component and determine factors influencing outcomes.

## Methods

### Source of data

All data were attained from a single major trauma centre, The Royal Infirmary of Edinburgh, between January 2009 and November 2012 and January 2018 to December 2019. All fractures were classified by the senior author (JFK) who has expertise in the management of these fractures, based on review of radiographs and CT imaging. Fractures involving the medial tibial plateau were first identified and categorized as either isolated medial plateau fractures or bicondylar fractures using both the Schatzker classification and the AO/OTA system. Within the cohort, 82 patients had Schatzker type IV medial plateau fractures and 61 had Schatzker type V/VI bicondylar fractures. According to the AO/OTA classification, these corresponded to 82 B-type (isolated medial plateau) fractures and 61 C-type (bicondylar) fractures.

Two separate databases were collected from consecutive series of tibial plateau fractures. This dual-cohort design was used to reflect evolving imaging and treatment practices over time. In the earlier cohort (2009 to 2012), CT imaging was not routinely employed, and fracture patterns were primarily assessed using plain radiographs, often leading to surgical management. This study took into consideration the 3D views seen on CT imaging based on the most up-to-date AO guidelines classification for tibial plateau fractures implemented in 2008, compared to the previously used 2D Schatzker classification. By the time of the later cohort (2018 to 2019), CT scans had become standard in tibial plateau fracture assessment throughout UK, allowing for more accurate classification. Importantly, 100% of patients in the later cohort and over 90% in the earlier cohort had CT imaging available. This improved imaging facilitated better understanding of fracture morphology, thereby enabling more nuanced decisions, including nonoperative management for certain stable fracture types. These advancements in diagnostic capability and evolving management strategies led to two separate datasets across different times. To ensure a uniform study population, patients were excluded if they were lost to follow-up, had incomplete electronic medical records, or were transferred to another hospital shortly after treatment.

### Patient characteristics

A total of 151 patients were initially enrolled in the study, of whom eight were lost to follow-up. This left a final cohort of 143 patients. These were categorized into two groups: those with isolated fractures of the medial tibial plateau (B-type fractures) and those with bicondylar patterns (C-type fractures). The aim was to compare outcomes and identify factors influencing results in both fracture types. The mean age was approximately 50 years for both fracture types (16 to 89). Mean follow-up duration differed: six months (0.5 to 33) for B-type and 8.7 months (0.5 to 46) for C-type fractures (Table I).

**Table I.** Basic demographic details and duration of follow-up.

Variable	AO/OTA B-type injury	AO/OTA C-type injury
Total, n (%)	82 (57)	61 (43)
Male, n (%)	37 (36)	36 (64)
Female, n (%)	45 (64)	25 (36)
Mean age, yrs (range)	50.3 (16 to 87)	49.5 (20 to 89)
Mean follow-up, mths (range)	6 (0.5 to 33)	8.7 (0.5 to 46)

OTA, Orthopaedic Trauma Association.

### Ethics and consent

This was a retrospective cohort study conducted using anonymized data obtained from electronic patient records. There was no direct interaction with patients, no interventions performed outside standard clinical care, and no disclosure of identifiable patient information. Consequently, individual informed consent was not required. The study protocol was approved by the Edinburgh Orthopaedic Department at Royal Infirmary of Edinburgh.

### Data collection

All electronic patient records were reviewed to obtain demographic details, mechanism of injury, major comorbidities, treatment provided, outcomes, duration of follow-up, and any additional interventions required. The mechanism of injury was categorized as either high-energy or low-energy trauma. High-energy trauma included mechanisms such as fall from height (FFH), fall downstairs (FDS), road traffic accidents (RTA), direct blow, assault, and crush injuries, whereas low-energy trauma referred to simple falls and sports-related injuries. Surgical management consisted of open reduction and internal fixation (ORIF), external fixation (ExFix), or total knee arthroplasty (TKA). Stable or undisplaced fractures treated conservatively were managed using a hinged knee brace for six to eight weeks. Outcomes and treatment-related complications were documented. Superficial infection was defined as clinical signs of infection resolving with oral antibiotics, while deep infection required revision surgery for abscess drainage or implant removal. Common peroneal nerve (CPN) palsy was diagnosed clinically, and compartment syndrome was identified through compartment pressure measurements and the requirement for fasciotomy. Post-traumatic osteoarthritis was diagnosed radiologically or by the need for TKA. Deep vein thrombosis (DVT) was confirmed using Doppler ultrasound. A comparison was then performed between OTA B-type and OTA C-type fractures to assess differences in outcomes.

### Statistical analysis

The Statistical Package for Social Sciences software v. 30 (IBM, USA) was used to analyze the collected data. Data are presented as means and percentages, unless otherwise specified. Parametric data were compared using the independent-samples *t*-test. Non-parametric complication data were analyzed using the chi-squared test. Logistic regression was conducted to explore the potential predictors of fracture type. Values of p < 0.05 were considered statistically significant.

## Results

### Demographic details

Out of 143 patients, 82 (57%) had B-type fractures, and 61 (43%) had C-type fractures. Poly-trauma was present in ten patients (12%) with B-type fractures and nine patients (15%) with C-type fractures. The cohort included 73 males and 70 females. Among the female patients, 45 (64%) had B-type and 25 (36%) had C-type fractures, while male distribution was nearly equal with 37 (36%) B-type and 36 (64%) C-type fractures (Table I).

### Mechanism of injury

Table II illustrates the distribution of the mechanism of injury (MOI) for B-type and C-type fractures. B-type fractures were more frequently associated with low-energy trauma (52.4%) compared with high-energy mechanisms (47.6%). In contrast, C-type fractures showed a higher proportion of high-energy trauma (59.0%) than low-energy causes (41.0%). Although the observed difference between the two fracture types was not statistically significant (p = 0.175, chi-squared test), the trend indicates that C-type fractures are more likely to result from high-energy trauma such as road traffic accidents or falls from height. This suggests that C-type fractures may be associated with more severe injury patterns; however, larger studies are necessary to confirm this association.

**Table II.** Mechanism of injury.

Injury mechanism	AO/OTA B-type injury	AO/OTA C-type injury
**Low-energy trauma**		
Simple fall	28	11
Sport	15	14
**High-energy trauma**		
FFH	15	13
RTA	11	14
FDS	10	6
Direct blow	3	1
Assault	0	1
Crush	0	1

FDS, fall downstairs; FFH, fall from height; OTA, Orthopaedic Trauma Association; RTA, road traffic accident.

### Comorbidities

As shown in Table III, the presence of comorbidities was more frequent in patients with C-type fractures compared to those with B-type fractures. Specifically, 63.9% of individuals in the C-type group (n = 38) had at least one comorbidity, whereas 51.2% of those in the B-type group (n = 42) were affected. Although this difference was not statistically significant (p = 0.129, chi-squared test), a trend towards a higher prevalence of comorbidities in the C-type group was observed. Additionally, when considering the cumulative presence of multiple comorbidities—such as smoking, alcohol consumption, diabetes, hypertension, and hypercholesterolaemia—a higher proportion was noted in the C-type group (82%) compared to the B-type group (65%). This suggests that patients with C-type fractures may be more medically complex. These comorbid conditions, particularly diabetes and obesity, could potentially contribute to increased complication rates, including infections. Therefore, future studies should account for comorbidities when evaluating clinical outcomes to better understand their influence on fracture management and prognosis.

**Table III.** Major comorbidities.

Major comorbidities	AO/OTA B-type injury	AO/OTA C-type injury
Smoking	24	24
Alcohol > 14 units per week	14	14
Diabetes	4	4
Hypertension	11	11
Hypercholesterolaemia	4	2
Total, %	65	82

OTA, Orthopaedic Trauma Association.

### Management

Table IV outlines the management strategies employed for B-type and C-type fractures, revealing that operative treatment was predominant in both groups. In the C-type group, 60 patients (98%) underwent surgical intervention, primarily through ORIF, with only one patient (2%) managed conservatively. Similarly, 74 patients with B-type fractures were largely treated with ORIF (89.4%), while eight patients (10.6%) received nonoperative management. The overall comparison between the two groups showed no statistically significant variance in treatment approach (p = 0.582), indicating that surgical management, particularly ORIF, was consistently preferred regardless of fracture type. This suggests that fracture classification does not significantly influence the decision to operate, as both B-type and C-type fractures were managed similarly.

**Table IV.** Mode of management.

Management	AO/OTA B-type injury	AO/OTA C-type injury
**Operative**		
ORIF	70	54
ExFix	1	0
ORIF + ExFix	1	4
Hinged TKA	2	2
**Nonoperative**	8	1
Conservative (knee brace)	8	1

ExFix, external fixation; ORIF, open reduction and internal fixation; OTA, Orthopaedic Trauma Association; TKA, total knee arthroplasty.

### Complications

As shown in Table V and Table VI, complication rates were slightly higher in the C-type fracture group, particularly for deep infections, compartment syndrome, and post-traumatic osteoarthritis. Overall, 27.9% of patients with C-type fractures experienced complications compared with 24.4% in the B-type group. Despite these differences, statistical analysis revealed no significant variation between the two groups (p = 0.639). These findings suggest that both fracture types carry a comparable risk of complications, with no clear evidence indicating a higher overall risk associated with either group.

**Table V.** Outcomes – infection and arthritic involvement.

Outcomes	AO/OTA B-type injury	AO/OTA C-type injury
**Superficial infection**		
Early	8	6
Late	3	0
**Deep infection**		
Early	4	7
Late	2	2
**Arthritic involvement**		
PTO	5	8
FFD	1	0
Varus deformity	3	4
Valgus deformity	0	1

Early (< 2 weeks), Late (> 2 weeks).

FFD, fixed flexion deformity; OTA, Orthopaedic Trauma Association; PTO, post-traumatic osteoarthritis.

**Table VI.** Outcomes – nerve injury and compartment syndrome.

Outcomes	AO/OTA B-type injury	AO/OTA C-type injury
**CPN palsy**		
Early	3	2
Late	1	0
**Compartment syndrome**		
Preop	0	2
Early	0	0
Late	0	0

Early (< 2 weeks), Late (> 2 weeks)

CPN, common peroneal nerve; OTA, Orthopaedic Trauma Association.

Nerve-related complications, such as common peroneal nerve (CPN) palsy, were rare in both groups—occurring in 4.9% of B-type and 3.3% of C-type fractures—with no statistically significant difference (p = 0.637, chi-squared test). Vascular and compartment-related issues showed a more distinct pattern. Compartment syndrome was observed exclusively in the C-type group (3.3%) and was absent in B-type fractures. Although not statistically significant (p = 0.099, chi-squared test), this finding is clinically relevant, as high-energy trauma is more common in C-type fractures and is known to increase the risk of compartment syndrome. DVT was infrequent in both groups, with a slightly higher incidence in B-type fractures (2.4%) compared with none in C-type (p = 0.219, chi-squared test).

Superficial infections were marginally higher in B-type fractures (13.4% vs 9.8%; p = 0.513, chi-squared test), while deep infections were nearly three times more common in C-type fractures (13.1% vs 4.9%; p = 0.079), nearing statistical significance. Post-traumatic osteoarthritis was more frequent in C-type fractures (13.1% vs 8.5%; p = 0.377), though not significantly. While most specific complications did not show significant differences between groups, C-type fractures demonstrated a trend towards higher rates of compartment syndrome and deep infections. Nerve injuries, DVT, and superficial infections were comparable between the two groups.

**Fig. 1:**
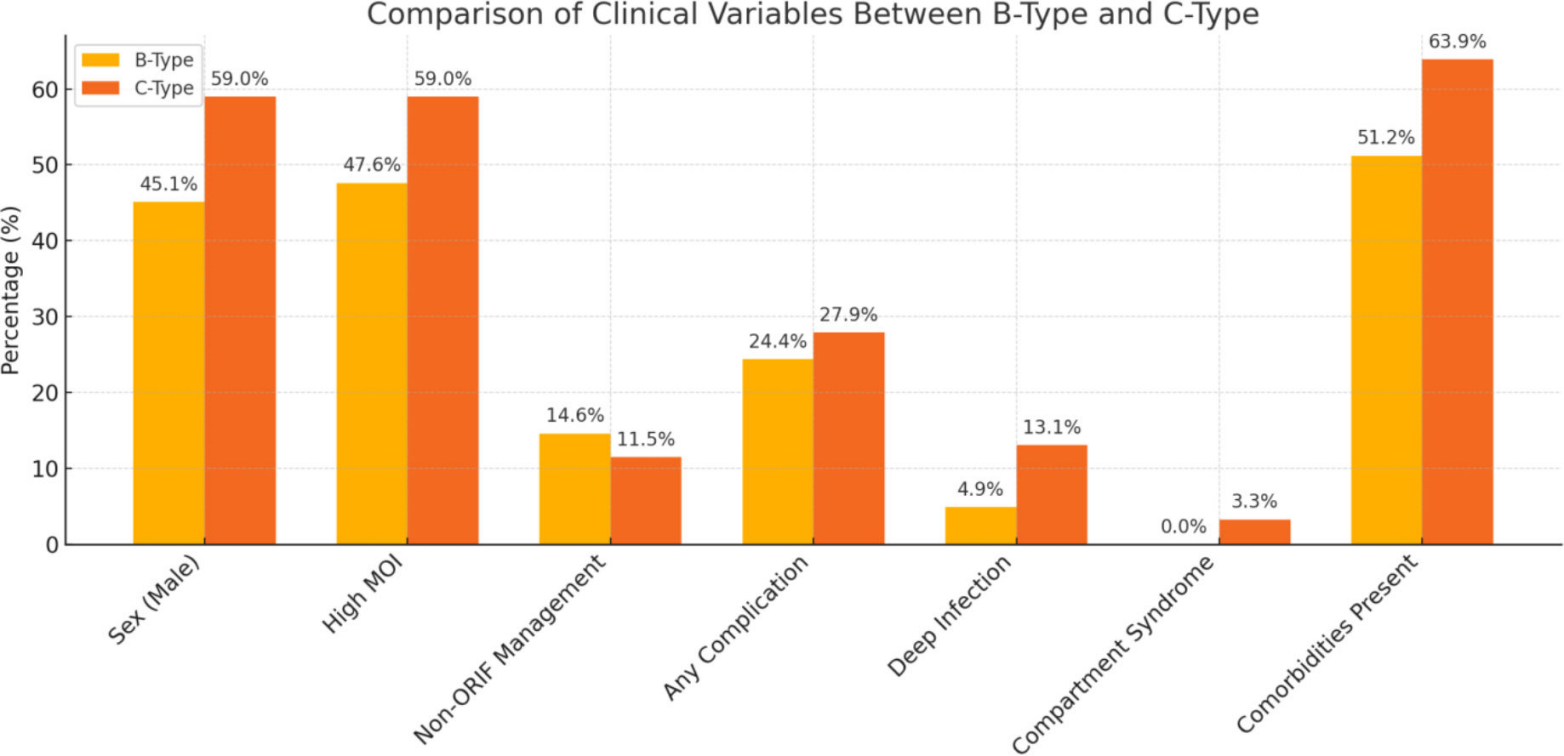
Percentage comparison of various clinical variables between B-type and C-type injuries. MOI, mechanism of injury; ORIF, open reduction and infection.

Comparison of the key variable among the two fracture types showed that there were no statistically significant differences between B-type and C-type fractures across the compared variables. However, deep infections (13.1% vs 4.9%, p = 0.079) and compartment syndrome (3.3% vs 0%, p = 0.099) were observed more frequently in C-type fractures, indicating a trend towards higher complication rates in this group. A bar chart visually compares the percentages and mean values for the variables listed in Table VII. Trends in complication rates and comorbidities appear elevated in the C-type group, although not to a statistically significant extent (Figure 1).

**Table VII.** Key variables compared between B-type and C-type fractures.

Variable	B-type (n = 82)	C-type (n = 61)	p-value	Odds ratio (Exp(B))	95% CI
Male sex, n (%)	37 (45.1)	36 (59.0)	0.100	1.76	0.90 to 3.45
Mean age, yrs (SD)	50.73 (18.62)	49.52 (16.94)	0.691	NS	NS
High-energy MOI, n (%)	39 (47.6)	36 (59.0)	0.175	1.58	0.81 to 3.07
**Management, n (%)**					
ORIF	70 (85.4)	54 (88.5)	0.582	1.00 (ref)	NS
Non-ORIF	12 (14.6)	7 (11.5)	NS	0.76	0.28 to 2.06
Any complication	20 (24.4)	17 (27.9)	0.639	1.19	0.57 to 2.49
Deep infection	4 (4.9)	8 (13.1)	0.079	2.92	0.85 to 10.05
Compartment syndrome	0 (0)	2 (3.3)	0.099	NS	NS
Comorbidities	42 (51.2)	39 (63.9)	0.129	1.70	N/A

MOI, mechanism of injury; N/A, not applicable; NS, not significant; ORIF, open reduction and internal fixation.

To further evaluate predictors of fracture type, a binary logistic regression analysis was performed using mechanism of injury, management approach, and complication occurrence as independent variables (Table VIII). The model demonstrated poor predictive accuracy, explaining only 5% of the variance, and failed to identify any statistically significant predictors. Among the variables, deep infection showed an odds ratio (OR) of 3.339, indicating that patients with deep infections had approximately 3.3 times higher odds of having a C-type fracture, although this was not statistically significant. Similarly, management with ORIF was associated with a slightly lower likelihood of C-type fractures (OR = 0.695), but again without statistical significance. These findings suggest that the variables assessed in this model offer limited ability to differentiate between B-type and C-type fractures. Further research is needed, incorporating more detailed anatomical features, soft-tissue involvement, or advanced predictive modelling techniques, to improve classification and understanding of fracture patterns.

**Table VIII.** Logistic regression results (mechanism of injury plus management plus complications).

Predictor	Odds ratio (Exp(B))	p-value	95% CI
High MOI	1.58	0.175	0.81 to 3.07
Non-ORIF management	0.76	0.582	0.28 to 2.06
Any complication	1.19	0.639	0.57 to 2.49
Constant	0.65	0.340	-

MOI, mechanism of injury; ORIF, open reduction and internal fixation.

## Discussion

In this study, outcomes following isolated medial tibial plateau fractures were no different from medial plateau fractures occurring in association with bicondylar fracture patterns. There was a definite trend for a higher rate of complications in the C-type bicondylar fracture patterns. However, with the numbers in the present study these differences did not achieve statistical significance. Medial plateau fractures are considered to be higher-energy injuries than isolated lateral tibial plateau fractures, and our findings would seem to confirm this. Essentially, the majority of medial plateau fractures can be considered higher-energy injuries with outcomes equivalent to the more complex bicondylar C patterns. It is generally assumed that fractures of the medial plateau are associated with increased risk of complications in comparison to the lateral plateau,^5^ although studies to confirm this are few.^6^ To our knowledge, this represents a large cohort study in a major trauma centre for isolated medial tibial plateau fractures with a long duration of follow-up.

The main demographic features of the two groups were also well matched, so there does not appear to be an influence on outcome of any of these characteristics. This brings our study’s focus onto providing a perspective on various factors that could potentially influence the outcomes of B-type and C-type fractures. Extensive research has been carried out to determine the correlation between the age of patients and its ability to affect the outcome of its fracture type. As described in several papers, the outcomes of either fracture groups remain similar, when patient age is taken into consideration. The majority of patients on the age spectrum demonstrated similar outcomes in B-type and C-type fractures that were not influenced by their age. Given this fact, we believe our results show that an average age of 50 years in both fracture groups has not played a role in the outcome of either fracture type.^7,8^ The mechanism of injury between both groups are different in nature, with B-type fractures occurring due to lower-energy trauma and C-type fractures taking place under higher energy.^3^ However, this does not discount the fact that isolated medial plateau fractures are of a high-energy trauma, in comparison to isolated lateral plateau fractures, which require a lower energy, because of the medial plateau’s thicker structure. We are comparing the B-type (isolated medial) and C-type (bicondylar) which, on review, would require a higher mechanism of injury to sustain this fracture pattern. A varus force onto an extended knee was mainly involved with B-type fractures, while a C-type fracture pattern was caused by valgus force with axial load sustained to a knee in extension.^9,10^ Our study found a similar correlation between the mechanism of injury of the two fracture types, in keeping with current literature. The findings of our study align closely with those reported by Cruz et al,^11^ who showed that outcomes in isolated medial tibial plateau fractures are primarily influenced by the underlying fracture morphology rather than broad classification. Their work demonstrated that medial plateau injuries, even when isolated, frequently result from high-energy mechanisms, reinforcing our observation that B-type fractures behave as high-energy injuries comparable to C-type patterns. The results from our study found that both fracture types had similar ratios of patients requiring surgical treatment with ORIF. Additionally, 10% of B-type fracture patients in this study group were amenable to conservative management compared with previous studies managing all isolated medial tibial plateau fracture patterns operatively. The study by Cruz et al^11^ further affirmed that ORIF continues to be the mainstay of treatment for medial plateau injuries, with surgical outcomes largely determined by restoration of joint morphology rather than fracture type, which is consistent with our observation. The most likely implication is that B-type fractures, which occur from high-energy injuries and have a more stable fracture pattern with minimal displacement, are well-suited for non-surgical treatment. This is consistent with the stronger subchondral bone on the medial side of the tibial plateau compared with the lateral condyle.

Studies conducted on treatment outcomes for tibial plateau fractures have demonstrated that surgical fixation is well established to have a satisfactory outcome for these fracture types.^2,12^ Rosteius et al^13^ demonstrated that outcomes in OTA B-type and C-type tibial plateau fractures depend more on the quality of articular reduction than on the fracture classification itself, with both types showing comparable results when joint congruity is restored. Their observation that surgical management is largely consistent across fracture types reinforces our conclusion that fracture type alone is not a reliable predictor of outcome, highlighting the greater importance of morphological factors and reduction quality. With various existing papers identifying numerous factors involved in the outcomes of tibial plateau injury, not many studies assess the long-term follow-up outcomes of individuals with surgical treatment.^3,14,15^

Of the outcomes considered in our study, deep infection rates were the most common serious complication, often associated with poorer functional outcomes, particularly knee stiffness. The cadaveric findings by Kalhor et al^16^ demonstrate that the medial tibial periosteum has a more vulnerable vascular pattern, which may partly explain the trend towards higher deep infection and soft-tissue complications observed in C-type fractures requiring medial or dual plating. Although radiological evidence of post-traumatic osteoarthritis was observed in both groups, progression to total knee arthroplasty (TKA) was rare, with only four patients ultimately requiring arthroplasty. This aligns with previous studies reporting a low long-term incidence of TKA following tibial plateau fractures. Gupta et al^17^ recently reported that pre-existing knee osteoarthritis and severe joint depression, rather than fracture type alone, were significant predictors of TKA in patients aged over 60 years. Their findings underscore the importance of assessing baseline joint health and fracture morphology to better predict long-term outcomes. In our cohort, while C-type fractures had a slightly higher rate of TKA, the difference was minimal, further supporting the idea that other factors beyond fracture classification may influence the need for joint arthroplasty.

The need for a TKA following a tibial plateau fracture is relatively low.^6^ The overall prevalence of TKA after tibial plateau fractures is reported to be 3% to 7% over ten years, according to a study conducted in Edinburgh for end-stage post-traumatic osteoarthritis.^18^ This is in keeping with our present findings, bearing in mind that C-type fractures had a 1% higher incidence of patients requiring hinged TKA. We are unaware of studies comparing the difference of incidence between both fracture types. Although these findings are in keeping with the results of the present study, other complications all occurred less frequently, and compartment syndrome was only seen in C-type fractures. A retrospective review that was undertaken in Taiwan highlighted a higher incidence of compartment syndrome in Schatzker Type VI fractures, which considered the fracture pattern and mechanism of injury.^19^ The prevalence of compartment syndrome in fractures that involves the diaphysis of the tibia is known to be higher compared with other fractures of the tibia. This review demonstrated that most of their patients with compartment syndrome (30.4%) came from those with Schatzker Type VI fractures, which is similar to the result found in our study that indicates a higher rate of compartment syndrome seen in C-type fractures compared with B-type fractures.^20,21^ The literature covering CPN palsy in tibial plateau fractures is limited, and there are no data comparing the incidence of this complication between medial tibial plateau fractures and bicondylar C-type fractures.

This study has several important strengths, including a relatively large sample size of 143 patients. It also benefits from a long follow-up duration of more than two years, allowing meaningful capture of late complications such as post-traumatic osteoarthritis and the need for subsequent total knee arthroplasty. To our knowledge, this represents one of the largest UK-based series reporting outcomes of fractures involving the medial tibial plateau, contributing valuable comparative data on isolated medial (AO Type B) and bicondylar (AO Type C) fractures, an area with limited published evidence, using uniform clinical and radiological assessment. Importantly, the study demonstrates that fracture classification alone may not reliably predict complications, highlighting instead the relevance of reduction quality, comorbidity burden, and fracture morphology in guiding management and prognosis.

It is always important to acknowledge certain limitations. The retrospective design, which relies on the accuracy and completeness of electronic patient records, may introduce information bias. Functional outcomes were not assessed using validated scoring systems, limiting the ability to correlate radiological or clinical findings with long-term functional recovery. Although fractures were classified using Schatzker and AO/OTA systems, the absence of detailed fracture morphology parameters and validated functional outcomes further restricts interpretation. The cohort included both isolated medial (B-type) and more complex bicondylar (C-type) fractures, creating heterogeneity that may have contributed to the lack of significant differences and the limited performance of the regression model. Finally, as a single major trauma centre retrospective cohort, the generalizability of these findings is limited.

In conclusion, based on these results, surgeons can be advised that the outcomes of isolated medial tibial plateau fractures are similar to bicondylar tibial plateau fractures in terms of complication rates. C-type fractures showed trends towards higher-energy trauma, greater comorbidity burden, and complications such as deep infection and compartment syndrome, although these were not statistically significant. One would generally assume that the isolated medial plateau fractures are less severe than bicondylar plateau fractures but, as it turns out, the medial plateau fracture which does tend to have a higher-energy pattern of injury has about the same outcome. As such, the outcomes of the isolated medial tibial plateau fractures are in fact equivalent to those of bicondylar tibial plateau fractures.

**Take home message**

- Isolated medial (AO Type B) tibial plateau fractures have comparable complication rates and overall outcomes to bicondylar (AO Type C) fractures, challenging the assumption that they are less severe injuries.

- Although C-type fractures demonstrated trends toward higher-energy mechanisms, deep infection, and compartment syndrome, these differences were not statistically significant.

- Fracture classification alone does not reliably predict outcome; reduction quality, fracture morphology, and patient comorbidities likely play a greater role in prognosis and management decisions.

## Data Availability

The data that support the findings for this study are available to other researchers from the corrsponding upon reasonable request.
